# 肺癌的性别差异及机制

**DOI:** 10.3779/j.issn.1009-3419.2011.07.12

**Published:** 2011-07-20

**Authors:** 昕 邢, 永德 廖, 和孝 唐, 广 陈, 晟 具, 良琨 游

**Affiliations:** 1 430030 武汉，华中科技大学同济医学院附属同济医院普胸外科 Department of General Thoracic Surgery, Tongji Hospital, Tongji Medical College, Huazhong University of Science and Technology, Wuhan 430030, China; 2 430033 武汉，湖北省中山医院心胸外科 Department of Thoracic Surgery, Hubei Zhongshan Hospital, Wuhan 430033, China

**Keywords:** 肺肿瘤, 性别, 差异, Lung neoplasms, Gender, Difference

## Abstract

肺癌是全球发病率、死亡率最高、治疗效果差的恶性肿瘤。肺癌在流行病学、病理类型、疗效和预后、甚至发病机制等多方面均表现出明显性别差异。对这些差异的深入剖析能更好地认识男女性别肺癌各自的特点，为肺癌防治采用不同的性别化措施提供新线索和思路；而对导致性别差异的具体机制进行深入研究，有助于阐明肺癌的发病机制。

## Abstract (en)

Lung cancer has been viewed as the most common malignant cancer with high incidence, mortality and poor survival all over the world. A lot of investigations indicated there are signifcant gender-associated differences in lung cancer in several characteristics such as epidemiology, pathology, clinical outcome and prognosis. The insight into these differences may help to clarify the gender-associated characteristics of lung cancer, and to drawn out new approach for treatment and prevent of lung cancer depending on gender-associated characteristics. Furthmore, study on mechanism of gender-associated characteristics may even help to illuminate the pathogenesis of lung cancer.

肺癌是目前最常见、死亡率最高的恶性肿瘤^[1]^，2010年美国癌症死因统计显示肺癌的死亡率在恶性肿瘤中居第一位^[2]^。肺癌的防治是全世界共同面对的一项难题，对肺癌患者实行个体化防治是目前研究的热点，而性别化防治是个体化防治的一个重要方面。研究发现肺癌的发病率、病理类型、发病机制、治疗效果及预后等方面存在明显的性别差异，但导致这些差异的具体机制尚不清楚，深入剖析这些差异并研究其机制，可为肺癌防治的性别化提供理论依据。

## 1 流行病学和病理类型

### 1.1 肺癌发病率的性别差异

以往肺癌多发生于男性，故被称作“男性癌”。但从近几十年的发病趋势来看，女性肺癌在世界范围均有升高。有报道^[3]^2000年-2005年中国男性肺癌发病数增加了26.9%，女性肺癌增加了30.5%，虽然女性肺癌发病数量较男性增长快，但目前男性肺癌发病率仍高于女性。近年全世界调查研究的多数结果显示男性总体肺癌发病率超过女性，男女之比为1.5:1-20:1。有研究^[4]^甚至发现男性肺癌发病率是女性的6倍。最近在西班牙的一项调查^[5]^也发现，45岁-64岁肺癌患者中男性是女性的4.6倍；在年龄较大的患者中该差异更明显，年龄越高，男性患者越多^[6]^。Radzikowska等^[4]^对20, 561例肺癌患者研究发现，女性肺癌的发病年龄小于男性（平均年龄：女性60岁*vs*男性62岁），年轻肺癌患者（< 50岁）中女性较男性多见，女性年轻患者占女性肺癌总数23.3%，男性年轻患者占男性肺癌总数12.6%。

肺癌发病率趋势也有性别差异，女性的发病率增长速度大于男性。国外调查^[7]^发现总体上肺癌发病率男性大于女性，男性肺癌发病率从1975年的90/10万变为2007年的85/10万，女性肺癌发病率从1975年的25/10万增加到2007年的55/10万，男性肺癌发病率增长较少甚至略有下降。我国男性肺癌发病率增长趋势从20世纪90年代起略有下降，而女性的发病率有上升趋势^[8]^。

总体上男性肺癌的发病率比女性高，并且在年龄高的人群中更明显。但在年轻人群中，女性肺癌的发病率则高于男性。从发病趋势看男性肺癌的发病率趋于平稳或略有下降，而女性肺癌的发病率有上升趋势。

### 1.2 肺癌危险因素的性别差异

可能存在性别差异的肺癌危险因素包括吸烟、空气污染以及肺癌家族史。

吸烟是肺癌重要的危险因素，但吸烟致肺癌存在性别差异。女性吸烟发生肺癌的危险性是否比男性高一直受关注。20世纪60年代起就相继有报道显示男性吸烟患肺癌的危险性大于女性，但近年较多的流行病学调查^[4]^却发现女性吸烟患肺癌的危险性大于男性。女性对烟草致癌化合物更敏感，相同暴露情况下女性比男性更易患肺癌等^[9]^。这可能与女性体内雌激素水平较高而促进肺癌进展有关^[10]^。近年女性吸烟数量的增加以及家庭内长期被动吸烟的存在可能是女性肺癌发病率增高的一个重要原因。

肺癌与空气污染密切相关，其中油烟污染在中国家庭中非常普遍，肺癌患者有油烟接触史的比例女性高于男性^[11]^。这可能是中国非吸烟女性患肺癌的重要原因之一。国外对生活燃料和烹饪油烟与肺癌危险关系的病例对照研究^[12]^也显示与女性有相关性，但与男性没有相关性。

家族史与肺癌发病率的关系越来越受到重视，研究发现肺癌家族史与其发病率存在性别差异。Nitadori等^[13]^在日本的一项前瞻性研究发现，一级亲属肺癌家族史阳性者患肺癌的相对危险度达到1.95，其中女性相对危险度为2.65，男性为1.69。我国的研究^[11]^也发现女性肺癌患者有肿瘤家族史的比例高于男性患者。肺癌在家族遗传方面所表现出的性别差异，可能是肺癌发病率性别差异的原因之一。

吸烟、油烟污染与肺癌家族史等危险因素表现出明显的性别差异，女性危险性大于男性，这些因素可能是导致女性发病率增加的原因。

### 1.3 肺癌病理类型的性别差异

鳞癌和腺癌是肺癌最常见的病理类型，且存在性别差异。鳞癌男性多见，腺癌女性多见。美国以往调查表明20世纪70年代早期男性肺癌发病率鳞癌是腺癌的2倍多，而女性发病率腺癌比鳞癌多30%。肺癌病理类型性别差异的研究包括Minami^[14]^、Cerfolio等^[15]^在内的较多研究均与以往的结论相同，女性比男性更容易患腺癌。目前肺腺癌已成为最常见的肺癌类型之一，约占所有肺癌的30%，且发病率近年在世界范围内呈明显上升趋势^[16]^。Alberg等^[17]^在调查美国1973年-1998年肺癌不同组织学类型发病率的变化趋势后发现，男、女性肺腺癌的标化发病率均明显增加，鳞癌男性在下降，女性在增加。虽然吸烟和肺鳞癌的相关性较大，但目前有一定的证据^[4, 15]^表明吸烟女性更容易患腺癌，特别是在青年女性肺癌患者，而且病变发展更快。

从上述资料看，男性肺癌以鳞癌为主，但鳞癌的发病趋势在下降而腺癌在上升，且腺癌的增长速度较快；女性肺癌主要病理类型是腺癌，而且其发病率仍在增长。目前关于肺腺癌发病率上升的原因尚不清楚，可能与几个因素有关^[18]^，如卷烟生产的变化包括低焦油含量、滤过嘴等导致肺癌组织学类型的改变；同时也可能与生活方式有关，如高脂膳食可能对肺癌类型产生影响^[19]^。

## 2 疗效和预后

目前认为肺癌的临床表现没有性别差异，但疗效和预后存在性别差异。

### 2.1 肺癌疗效的性别差异

肺癌的治疗仍以手术切除为主，此外还有化疗、放疗和靶向治疗等。在手术治疗方面，有研究^[11]^发现不可手术的患者（Ⅲb期和Ⅳ期）女性多于男性，可能与女性腺癌比例高有关。肺腺癌多为周围型，早期症状少且容易广泛转移，从而导致就诊时分期较晚。该研究同时也发现，有胸腔积液的不可手术患者女性多于男性，这也可能与女性腺癌多、容易侵犯胸膜有关。

关于肺癌化疗效果多数研究发现男女之间存在差异。Keller等^[20]^研究非小细胞肺癌化疗长期生存的原因显示，女性被视为有利预后因素。Cerfolio等^[15]^、Yamamoto等^[21]^的研究也发现非小细胞肺癌化疗疗效女性优于男性。这也可能与女性易患腺癌有关。目前的研究^[22]^尚未发现放疗效果存在性别差异。

肺癌分子靶向治疗是近年来发展起来的肺癌治疗方法，治疗效果存在性别差异。目前以表皮生长因子受体（Epidermal growth factor receptor, EGFR）为靶点的肺癌靶向治疗研究最多也最有成效^[23]^，应用于临床治疗的主要靶向药物是吉非替尼，多数研究^[24]^提示治疗效果女性要好于男性，这可能与女性肺癌中EGFR基因的突变率比男性肺癌高有关。

### 2.2 肺癌生存率的性别差异

Asamura等^[25]^对13, 010例手术切除肺癌的研究表明，男性和女性非小细胞肺癌的5年生存率分别是14.8%和20.3%，男性和女性小细胞肺癌的5年生存率分别是5.1%和7.1%，女性均高于男性。国内的研究^[26]^也发现完全切除术后女性患者生存率高于男性。对不同分期肺癌预后的研究^[16]^也提示非小细胞肺癌Ⅰ期-Ⅲ期的5年生存率无论总体还是各个分期女性均高于男性。Doning等^[27]^研究发现女性肺癌的预后比男性好、生存期长，其原因可能与女性患者对治疗的敏感性高有关。以上资料均表明，肺癌患者的预后女性优于男性。然而也有少数不同观点，如Jubelirer等^[28]^对2, 207例早期肺癌切除术后的患者的回顾性研究发现肺癌患者的5年生存率不存在性别差异。这与早期肺癌患者手术效果和预后好，5年生存率均较高有关，综合分析迄今大多数研究结果女性肺癌患者生存率高于男性。

## 3 肺癌性别差异机制的探讨

肺癌的发生发展与环境、遗传和内分泌等诸多因素密切相关，这些致病因素作用于不同性别人群产生的效果不同，这可能是导致肺癌性别差异的原因，但确切的生物学机制尚不清楚。

### 3.1 吸烟与肺癌的性别差异

吸烟是肺癌重要的致病因素，据估计有80%-90%的肺癌与吸烟或被动吸烟有关，多数研究结果表明吸烟与肺癌的相关性女性比男性大。导致该差异的机制尚不清楚，目前研究提示可能与基因和烟草致癌物代谢有关。

在基因方面，可能与肺癌中p53抑癌基因的突变、*K-ras*原癌基因的启动以及基因启动子甲基化不同有关^[29]^。研究^[30]^发现女性肺癌从未吸烟者*p53*基因突变率为19%，而有吸烟史者突变率达67%；对吸烟者K-ras基因突变的研究^[31]^也发现女性比男性更常见；启动子甲基化使基因转录活性被抑制，对肺癌启动子甲基化活性的研究^[32, 33]^发现，男性吸烟者启动子甲基化比女性明显增高，这些可能是导致女性吸烟者更易患肺癌的重要机制。

在烟草致癌物代谢方面，可能与谷胱甘肽S-转移酶（glutathione S-transferases, GSTs）、多环芳香烃（polycyclic aromatic hydrocarbons, PAH）和DNA加合物有关。GST^[34]^是烟草致癌物代谢的第二阶段酶类，其中与烟草致癌物代谢活性有关的有GSTM1。GSTM1阴性表达患肺癌的风险更高^[35, 36]^，吸烟者中女性阴性表达比男性高，因而更易患肺癌。PAH也是严重的致癌物，PAH加合物水平在女性体内更高。烟草致癌物损伤DNA，在DNA修复过程中如果形成DNA加合物，则容易导致肺癌的发生^[37, 38]^；女性吸烟者体内DNA加合物水平比男性更高，因此，吸烟的女性更容易发生肺癌。

### 3.2 雌激素与肺癌的性别差异

近年越来越多的研究提示雌激素在肺癌的发生发展中可能起重要作用，表现为雌激素替代治疗明显增加女性患肺癌的危险并且降低生存率^[39]^、非小细胞肺癌患者外周血中雌激素水平较健康者明显增高^[40]^、肺癌局部组织中雌激素水平较正常肺组织高^[41]^、肺癌组织过表达雌激素受体、雌激素能明显促进肺癌细胞增殖、在鼠肺癌模型中雌激素促进肺癌的发生^[42]^、雌激素受体的亚细胞定位和活性有明显的性别差异^[43]^。性激素是男女性最主要的差异之一，因此雌激素很可能是导致肺癌性别差异的重要机制，但尚需进一步研究证实。

雌激素主要通过雌激素受体发挥作用。雌激素受体（Estrogen receptor, ER）有两种亚型：ERα和ERβ。大量研究^[44-48]^表明ERβ在肺癌组织中的表达明显高于正常肺组织，很可能是肺癌中的主要功能受体。有研究^[42, 45]^发现ERβ的表达、亚细胞定位和活性有明显的性别差异，不同性别来源的肺癌细胞株对雌激素的反应不同。这些可能是肺癌性别差异的重要机制，但尚有待大量研究证实。关于ERβ在肺癌组织中表达的性别差异，其研究结果并不统一^[45, 46]^。有研究^[46]^表明肺癌组织中ERβ表达不存在性别差异。近年来关于ERβ研究的最新突破是ERβ亚型ERβ1-5的鉴定^[47, 48]^，不同病例中所起主导作用的ERβ亚型可能不同^[49, 50]^，ERβ1-5可能成为研究雌激素与肺癌性别差异的重要突破口。

以上资料表明，雌激素对肺癌的发生发展可能起重要作用，其受体的表达、定位和活性可能存在性别差异，可能是导致肺癌性别差异的重要机制，深入研究将有利于阐明雌激素促肺癌的作用和机制，为肺癌性别化防治提供依据和切入点。

### 3.3 遗传易感性与肺癌的性别差异

近年来对肺癌遗传易感性的研究是肺癌病因学研究的热点。与肺癌相关的遗传因素较多，但其中存在性别差异的因素有DNA损伤修复基因的多态性、细胞色素氧化酶P450（CYP450）家族基因的多态性和*EGFR*基因突变等。

DNA损伤修复基因在维持基因组功能整体性、修复致癌因素导致的损伤和抗癌过程中有着重要的作用。其中X射线损伤修复交叉互补基因（X-ray repair cross-complementing, XRCC）的多态性与肺癌的关联可能存在性别差异。*XRCC1*基因Arg399Gln多态性可能与吸烟男性肺癌有明显的相关性，Ryk等^[51]^的研究发现具有Arg399Gln基因型的吸烟男性患肺癌风险明显增加，并且男性是女性的4倍。也有研究^[52]^表明Arg399Gln多态性可能是非吸烟女性患肺腺癌的遗传易感因素。

CYP450家族基因的表达和多态性与肿瘤易感性相关联，其中在肺癌易感性中起决定作用的*CYP1A1*基因表达和多态性可能存在性别差异。女性*CYP1A1*基因表达患肺癌风险将比男性更高^[53]^。Mollerup等^[54]^的研究发现女性*CYP1A1*基因表达水平比男性高2.4倍。因此，女性比男性患肺癌的风险更高。研究^[55]^显示*CYP1A1*基因m2位点多态性增加中国女性肺癌易感性，在非吸烟女性中更明显，但并不增加男性发生肺癌的风险。

EGFR是第一个被发现的由原癌基因编码的酪氨酸激酶erbB家族成员。目前研究^[56]^表明肺癌EGFR表达存在性别差异，女性患者EGFR基因突变更常见。这些被认为是导致EGFR抑制剂吉非替尼治疗肺癌临床疗效女性优于男性的重要机制，也是人类探讨肺癌性别化治疗的成功范例。

### 3.4 病毒感染与肺癌的性别差异

病毒感染与肺癌的相关性近年来逐渐受到关注，与肺癌相关的病毒有人类乳头瘤病毒（human papillomavirus, HPV）、人类免疫缺陷病毒、流感病毒等，其中存在性别差异的肺癌相关性病毒是HPV。

HPV感染几乎是所有宫颈癌（95%-100%）的致病因素^[57]^，特别是HPV16、HPV18风险性更高。由于在肺癌癌前病变和癌组织能检测到HPV，由此推测HPV感染与肺癌相关^[58, 59]^。流行病学调查发现^[60, 61]^HPV感染相关的肺腺癌发病率女性更高，这可能与女性更容易感染和传播HPV并通过血液循环途径移行到肺部有关。迄今对HPV感染与肺癌的相关性机制还未阐明，有研究提示HPV感染可能是众多内外源性肺癌致病因素的促进因子，而非直接致癌因子。

目前关于肺癌性别差异的机制探讨已取得很大进展和突破，多数人认为吸烟、CYP450家族基因表达与多态性、HPV感染、EGFR表达等因素与女性肺癌的相关性大于男性，而XRCC1多态性等因素男性大于女性。雌激素及其受体可能是导致肺癌性别差异的重要因素，甚至是肺癌性别化防治的潜在靶点，仍需进一步探讨。

## 4 展望

肺癌是最常见的恶性肿瘤，大多数肺癌确诊时已到中晚期，治疗手段有限，治疗效果不佳。因此，肺癌的预防、治疗，特别是针对不同患者的个体化治疗将是今后研究工作的重点。肺癌性别差异的研究对于肺癌的防治和指导今后的研究方向均有意义。目前女性肺癌发病率在世界范围内呈上升趋势，女性肺癌中腺癌占大部分，就诊时多为晚期，不可手术切除比例较男性高，因此女性更应注意肺癌的预防和早期发现，尤其是针对存在肺癌高危险因素如吸烟、遗传、内分泌疾病和HPV感染的女性群体的防治是今后肺癌防治策略的重要方向。

肺癌性别差异机制的探讨是最终揭示肺癌性别差异的根本，也是肺癌性别化防治的基石。虽然雌激素对肺癌的发生发展可能起重要作用，但它是否是导致肺癌性别差异的重要因素尚有待更多的研究证实，该课题可能会成为未来研究的热点。

肺癌的分子靶向治疗是目前研究的热点，目前大多数研究^[62]^表明吉非替尼对肺癌有治疗效果，但疗效有很明显的性别差异。这提示今后肺癌药物的研究方向可根据肺癌的性别差异来研究药物。目前肺癌治疗效果女性优于男性，而男性肺癌的发病率较女性高，因此针对男性患者的靶向治疗的研究十分必要，更重要的是，研究这些性别差异可为实行肺癌防治的性别化提供理论依据。
